# Multilevel analysis of healthcare utilization for childhood diarrhea in high under five mortality countries

**DOI:** 10.1038/s41598-024-65860-1

**Published:** 2024-07-04

**Authors:** Misganaw Guadie Tiruneh, Melak Jejaw, Kaleb Assegid Demissie, Tesfahun Zemene Tafere, Demiss Mulatu Geberu, Asebe Hagos, Lemlem Daniel Baffa, Getachew Teshale

**Affiliations:** 1 Department of Health Systems and Policy, Institute of Public Health, College of Medicine and Health Sciences, University of Gondar, P.O. BOX 196, Gondar, Ethiopia; 2 Department of Human Nutrition, Institute of Public Health, College of Medicine and Health Sciences, University of Gondar, Gondar, Ethiopia

**Keywords:** DHS, Utilization, Healthcare, Children, Under-five mortality, Diseases, Health care, Medical research

## Abstract

Globally, 4.9 million under-five deaths occurred before celebrating their fifth birthday. Four in five under-five deaths were recorded in sub-Saharan Africa and Southern Asia. Childhood diarrhea is one of the leading causes of death and is accountable for killing around 443,832 children every year. Despite healthcare utilization for childhood diarrhea has a significant effect on the reduction of childhood mortality and morbidity, most children die due to delays in seeking healthcare. Therefore, this study aimed to assess healthcare utilization for childhood diarrhea in the top high under-five mortality countries. This study used secondary data from 2013/14 to 2019 demographic and health surveys of 4 top high under-five mortality countries. A total weighted sample of 7254 mothers of under-five children was included. A multilevel binary logistic regression was employed to identify the associated factors of healthcare utilization for childhood diarrhea. The statistical significance was declared at a *p*-value less than 0.05 with a 95% confidence interval. The overall magnitude of healthcare utilization for childhood diarrhea in the top high under-five mortality countries was 58.40% (95% CI 57.26%, 59.53%). Partner/husband educational status, household wealth index, media exposure, information about oral rehydration, and place of delivery were the positive while the number of living children were the negative predictors of healthcare utilization for childhood diarrhea in top high under-five mortality countries. Besides, living in different countries compared to Guinea was also an associated factor for healthcare utilization for childhood diarrhea. More than four in ten children didn’t receive health care for childhood diarrhea in top high under-five mortality countries. Thus, to increase healthcare utilization for childhood diarrhea, health managers and policymakers should develop strategies to improve the household wealth status for those with poor household wealth index. The decision-makers and program planners should also work on media exposure and increase access to education. Further research including the perceived severity of illness and ORS knowledge-related factors of healthcare utilization for childhood diarrhea should also be considered by other researchers.

## Introduction

Globally, 4.9 million under-five deaths (one child every six seconds) were recorded in 2022. Of the total 4.9 million under-five deaths, 2.3 million occurred during the first thirty days of life, and 2.6 million deaths happened between the ages of 1 and 59 months. Four in five under-five deaths occurred in sub-Saharan Africa and Southern Asia; they account for 57% and 26% of under-five deaths, respectively^1^. Although the death rate significantly declined by more than half from the 2000 report, further efforts are needed to accelerate progress towards the Sustainable Development Goal (SDG) target of 25 child deaths per 1000 live births by 2030^2^. Evidence showed that most under-five deaths in low-and middle-income countries are caused by preventable and treatable diseases such as diarrhea, fever, cough, pneumonia, and malaria^1,3^.

Childhood diarrhea is one of the leading causes of death and is responsible for killing around 443,832 children every year^3,4^. According to a UNICEF report in Mali, Niger, and Chad childhood diarrhea causes 18.5%, 17.5%, and 17.2% of child mortality, respectively^5^. In addition, 10.6% of child mortality in Guinea also due to the diarrheal diseases^5^. Despite healthcare utilization for childhood diarrhea having a significant effect on the reduction of childhood mortality and morbidity, most children die due to delays in seeking health care in developing countries^6–8^. Different studies reported that distance from healthcare facilities, limited healthcare access, poor knowledge about the symptoms of diseases, perceived curability of illness, lack of money, and a long period of waiting for medical services were the main barriers to low healthcare utilization in developing countries^6,7,9–12^.

Healthcare utilization for childhood diarrhea was low in developing countries^10,12–14^. For example, a study conducted in Nigeria showed that only 27% of children seek healthcare^14^. Correspondingly, in Ethiopia, the magnitude of healthcare utilization for childhood diarrhea was 35%^15^. Additionally, a systematic review conducted in sub-Saharan Africa also revealed that only 45% of children utilized healthcare for childhood illnesses^16^.

Literature showed that different factors affecting healthcare utilization for childhood diarrhea include place of residence^12,15,17,18^, maternal age^18–22^, maternal educational status^6,16,21–24^, sex of the child^22,25^, age of the child^6,15,19,22^, husband educational status^19,23^, marital status^17,19,26^, household wealth index ^12,18,19,21–25^, media exposure^16,18,22,25^, distance to health facilities^16,20,21,27^, information about oral rehydration^15,25^, place delivery^6,14^, and child birth order^23^.

Different studies related to healthcare utilization have been conducted at the country level^6,10,13,14,20,21,28^. However, there is no evidence of healthcare utilization for childhood diarrhea in top high under-five mortality countries. Therefore, this study aimed to generate evidence of healthcare utilization for childhood diarrhea in the top high under-five mortality countries by including DHS data from 2013/14 to 2019. The result will help to improve healthcare utilization for childhood diarrhea and to design an intervention strategy to address poor child health status and outcomes in high under-five mortality countries.

## Methods

### Study setting and design

The study used pooled data from the top high under-five child mortality countries Demographic and Health Survey (DHS) data collected between 2013/14 and 2019, which was obtained using a community-based cross-sectional study design. The countries identified as having the top ten highest under-five mortality rates were selected from the United Nations (UN) child mortality estimation report of 2023^1^. According to the UN report; Niger, Nigeria, Somalia, Chad, Sierra Leone, South Sudan, Central Africa Republic, Guinea, Mali, and DRC were the top ten high under-five mortality countries. Somalia and South Sudan were not included due to the lack of a DHS dataset. In addition, the Central African Republic and Niger were also excluded due to the long period since their last standard DHS (Table 1).

**Table 1:** Top ten highest child mortality countries with their respective DHS year.

Countries	Under-five mortality rate (deaths per 1,000 live births)	Latest DHS year
Niger	117	2012
Nigeria	107	2018
Somalia	106	No DHS dataset
Chad	103	2014/15
Sierra Leone	101	2019
South Sudan	99	No DHS dataset
Central Africa Republic	97	1993
Guinea	97	2018
Mali	94	2018
DRC	76	2014

### Data source and study population

**Figure 1:**
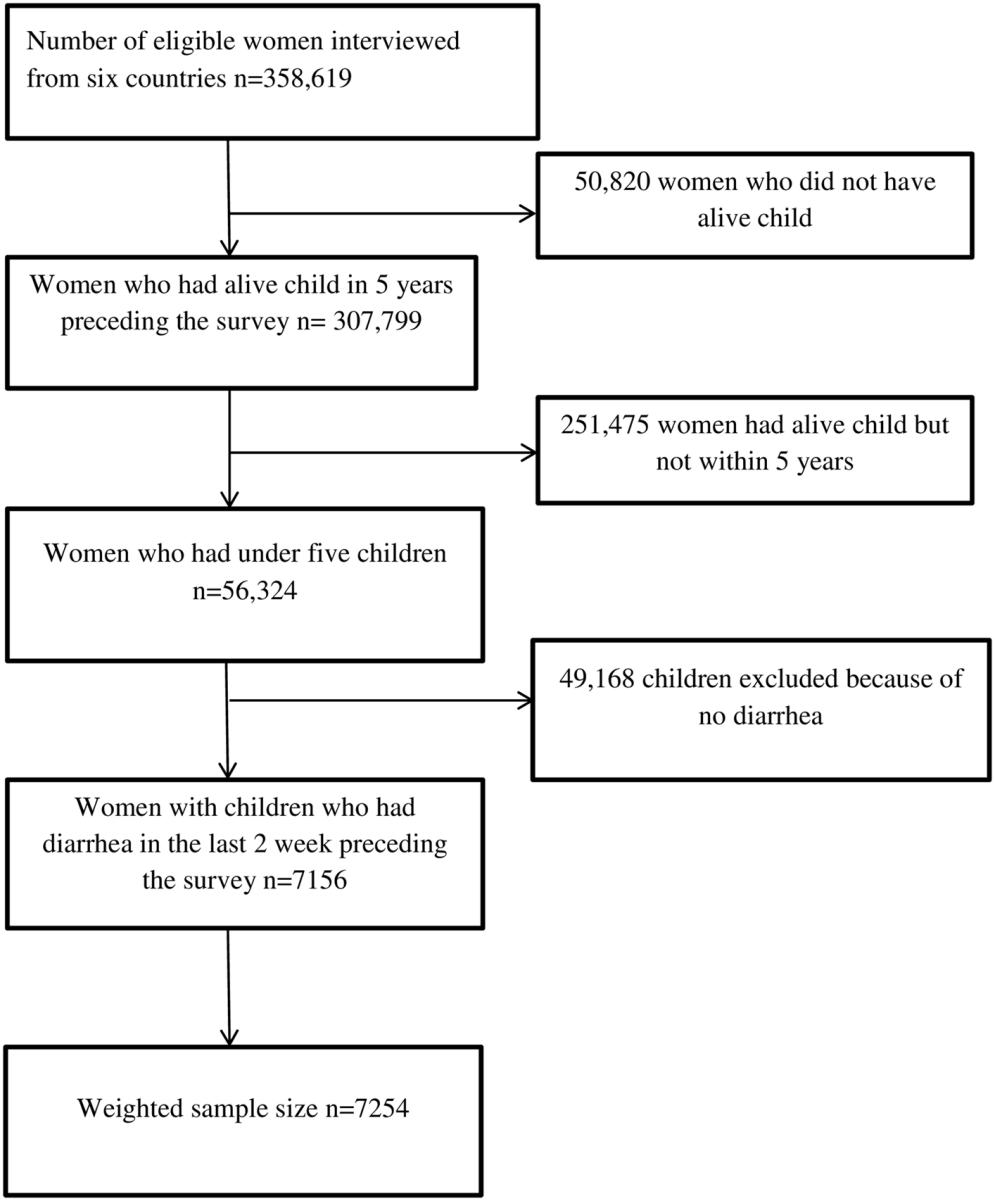
Final sample size and schematic presentation of how the study sample size was selected.

The analysis was based on the secondary data from the most recent DHS of the top high under-five mortality countries. The DHS program collects standard and comparable data in low-and middle-income countries. The surveys are nationally representative and population-based, with large sample sizes of the same manual, variable name, code, value level, and procedure in more than 90 countries across the world^29^. The survey used a two-stage stratified sampling technique every five years. In the first stage, enumeration area (EA) clusters were selected by the proportional sample size method. Then, a fixed number of households per cluster was selected by equal probability systematic sampling following the list of households^29^. The DHS data were collected using face-to-face interviews with reproductive-aged 15–49-year-old women. Detailed survey methodology and sampling methods used in gathering the data are available^29^. The surveys collect a wide range of self-reported and objective data, with a strong focus on indicators of maternal and child health, reproductive health, nutrition, fertility, mortality, and self-reported health behaviors among adults^30^. Before analysis, weighting was done to get a representative sample by dividing the individual weight for women (v005) by 1,000,000 to estimate the number of cases^29^. The total weighted sample size for this study was 7254, which included Guinea (1043), Mali (1631), Nigeria (3950), and Sierra Leone (630). Chad and the Democratic Republic Congo (DRC) were excluded after appending the data because they had no observation of the outcome variable. The study included children who had diarrhea in the 2 weeks preceding the surveys, whether they sought public or private healthcare or not (Fig. 1).

## Study variables

### Outcome variable

The outcome variable for this study was healthcare utilization for childhood diarrhea reported by the mother or caregiver. The DHS collected data on whether the child had diarrhea in the 2 weeks preceding the survey, and the healthcare utilization for childhood diarrhea was also assessed by interviewing the mothers. The mothers were considered to have utilized healthcare if they sought medical treatment from a defined governmental or non-governmental health facility for childhood diarrhea coded as “1”, and mothers not seeking healthcare were coded as “0”.

### Independent variables

We considered individual and community-level variables for this study. At the individual level; sex of the child, age of the child, maternal age, maternal educational status, husband’s educational status, current marital status, sex of household head, wealth index, media exposure, information about oral rehydration, covered by health insurance, wanted last child, place of delivery, birth order, and number of living children were included. At the community level, place of residence, distance to the health facility, community-level poverty, community-level media exposure, community-level education, and countries were considered.

Community-level variables used in the analysis were from two sources; direct community-level variables (including place of residence, country, and distance to health facility) that were used without any aggregation and aggregated community-level variables that were generated by aggregating individual-level variables at the cluster level. The community-level education, community-level poverty, and community-level media exposure were generated by aggregating the individual-level variables at the cluster level and categorized them as low if the proportion is < 50% and high if the proportion is ≥ 50% based on the national median value by considering their frequency distribution^31^.

### Media exposure

Was generated from the frequency of listening to the radio, watching television, and reading a newspaper or magazine. Respondents who never listened to the radio, read newspapers, or watched television were considered to have no exposure to mass media, and were otherwise exposed to mass media.

### Wealth index

The variable wealth index was re-categorized as “poor”, “middle”, and “rich” by merging poorest and poorer as “poor” and richest with richer as “rich”.

### Data collection procedure

The research was performed based on the DHS data by accessing it from the official database of the MEASURE DHS program www.measuredhs.com. For the study, we used the Birth Record (BR) data set file.

### Data management and analysis

The variables in this study were extracted and analyzed from the BR dataset using STATA version 17.0 (StataCorp, College Station, Texas 77,845 USA) statistical software which is available at https://www.stata.com. The extracted data from the included countries were weighted using sampling weight (v005) to obtain a valid statistical estimation. In DHS, multi-stage stratified cluster sampling techniques were employed, and the data were hierarchical. So, to draw valid inferences and conclusions, a multilevel model was fitted. A two-level binary logistic regression model was used to estimate the effect size of independent variables on healthcare utilization for childhood diarrhea. Four models were fitted. The first model was the null model (a model without the independent variable), which was a model fitted to calculate the extent of cluster variability on healthcare utilization for childhood diarrhea. It was assessed using the Intraclass Correlation Coefficient (ICC), Likelihood Ratio test (LR), Median Odds Ratio (MOR), and Proportional Change in Variance (PCV). The Intraclass Correlation Coefficient (ICC) was used to quantify the degree of heterogeneity of healthcare utilization for childhood diarrhea between clusters. The null model provides the variance of the outcome variable due to the cluster without the independent variables (to evaluate the extent of the cluster variation in healthcare utilization for childhood diarrhea). Considering clusters as a random variable, the MOR indicates the median value of the odds ratio between the area at the highest risk and the area at the lowest risk of healthcare utilization for childhood diarrhea when randomly picking out two different clusters. Proportional Change in Variation (PCV) was reported to assess the total variation of healthcare utilization for childhood diarrhea explained by the final model (a model with individual-level and community-level variables) relative to the null model (a model without explanatory variables). Model I (a model that includes only individual-level factors), model II (a model that includes only community-level factors), and model III (a model adjusted with both individual and community-level factors) were fitted, and a model comparison was made by using deviance.

Both bi-variable and multivariable analyses were done. In the bi-variable, two-level binary logistic regression analysis, variables with a *p*-value ≤ 0.2 were considered in the multivariable analysis. The Adjusted Odds Ratio (AOR) with a 95% Confidence Interval (CI) at *p*-value < 0.05 in the multivariable multilevel analysis was reported to declare the statistical significance and strength of the association between healthcare utilization for childhood diarrhea and independent variables. Before multi-variable analysis, multi-collinearity was checked using the Variance Inflation Factor (VIF), and the mean VIF was 2.02.

### Ethical approval

The data were accessed from the DHS website https://dhsprogram.com/data/available-datasets.cfm after getting registered and permission. The retrieved data were used for this registered research only. The data were kept confidential and no identifier was made to identify any household or individual respondent.

## Result

### Individual level characteristics

A total of 7254 mothers who have under-five children were included in this study. Out of the study participants, 3373 (46.50%) were aged between 25 and 34 years. Of the respondents, 4611 (63.57%) of them had no formal education, and the majority (94.95%) were in union in marital status. More than half (52.73%) of the participants were from poor household wealth status. Most (85.81%) of respondents had information about oral rehydration, but 97.58% of participants were not covered by health insurance. Of the involved children, nearly half (48.64%) of them were females, and 54.74% of them were less than two years old (Table 2).

**Table 2:** Individual level characteristics of mothers/caregivers of under-five children in top high under-five mortality countries.

Variables	Category	Weighted frequency (%)
Sex of child	Male	3726 (51.36%)
	Female	3528 (48.64%)
Age of the child	< 12 months	1710 (23.57%)
	≥ 12 months	5544 (76.43%)
Maternal age	15–24 years	2268 (31.27%)
	25–34 years	3373 (46.50%)
	35–49 years	1613 (22.23%)
Maternal educational status	No education	4611 (63.57%)
	Primary	1036 (14.28%)
	Secondary & above	1607 (22.15%)
Husband’s educational status	No education	4001 (58.09%)
	Primary education	764 (11.09%)
	Secondary & above	2123 (30.81%)
Current marital status	Never in union	183 (2.52%)
	In union	6888 (94.95%)
	Formerly in union	183 (2.52%)
Sex of household head	Male	6469 (89.18%)
	Female	785 (10.82%)
Wealth index	Poor	3825 (52.73%)
	Middle	1421 (19.59%)
	Rich	2008 (27.68%)
Media exposure	Exposed	4587 (63.24%)
	Non-exposed	2666 (36.76%)
Information about oral rehydration	Yes	6225 (85.81%)
	No	1029 (14.19%)
Covered by health insurance	Yes	175 (2.42%)
	No	7078 (97.58%)
Wanted last-child	Wanted	7066 (97.41%)
	Unwanted	188 (2.59%)
Place delivery	Home	3948 (54.42%)
	Health facility	3306 (45.58%)
Birth order	First	1327 (18.30%)
	Second	1286 (17.73%)
	Third	1086 (14.97%)
	Fourth or more	3555 (49.00%)
Number of living children	1_2 children	2688 (37.06%)
	3_4 children	2349 (32.38%)
	5 + children	2217 (30.56%)

### Community level characteristics

Of the participants, 72.63% of mothers/caregivers were rural dwellers. About half of the participants were from communities with a high proportion of community-level poverty and a low proportion of community-level education. The highest number of participants was from Nigeria, 3950 (54.45%), and the lowest number of study participants was from Sierra Leone, 630 (8.68%) (Table 3).

**Table 3:** Community-level characteristics of mothers/caregivers of under-five children in top under-five mortality countries.

Variables	Category	Weighted frequency (%)
Place of residence	Urban	1985 (27.37%)
	Rural	5269 (72.63%)
Distance to the health facility	Big problem	2644 (36.45%)
	Not big problem	4610 (63.55%)
Community level education	High	3560 (49.07%)
	Low	3694 (50.93%)
Community level poverty	High	3637 (50.14%)
	Low	3617 (49.86%)
Community-level media exposure	High	3819 (52.65%)
	Low	3435 (47.35%)
Country of residence	Guinea	1043 (14.37%)
	Mali	1631 (22.48%)
	Nigeria	3950 (54.45%)
	Sierra Leone	630 (8.68%)

### Healthcare utilization for childhood diarrhea

**Figure 2:**
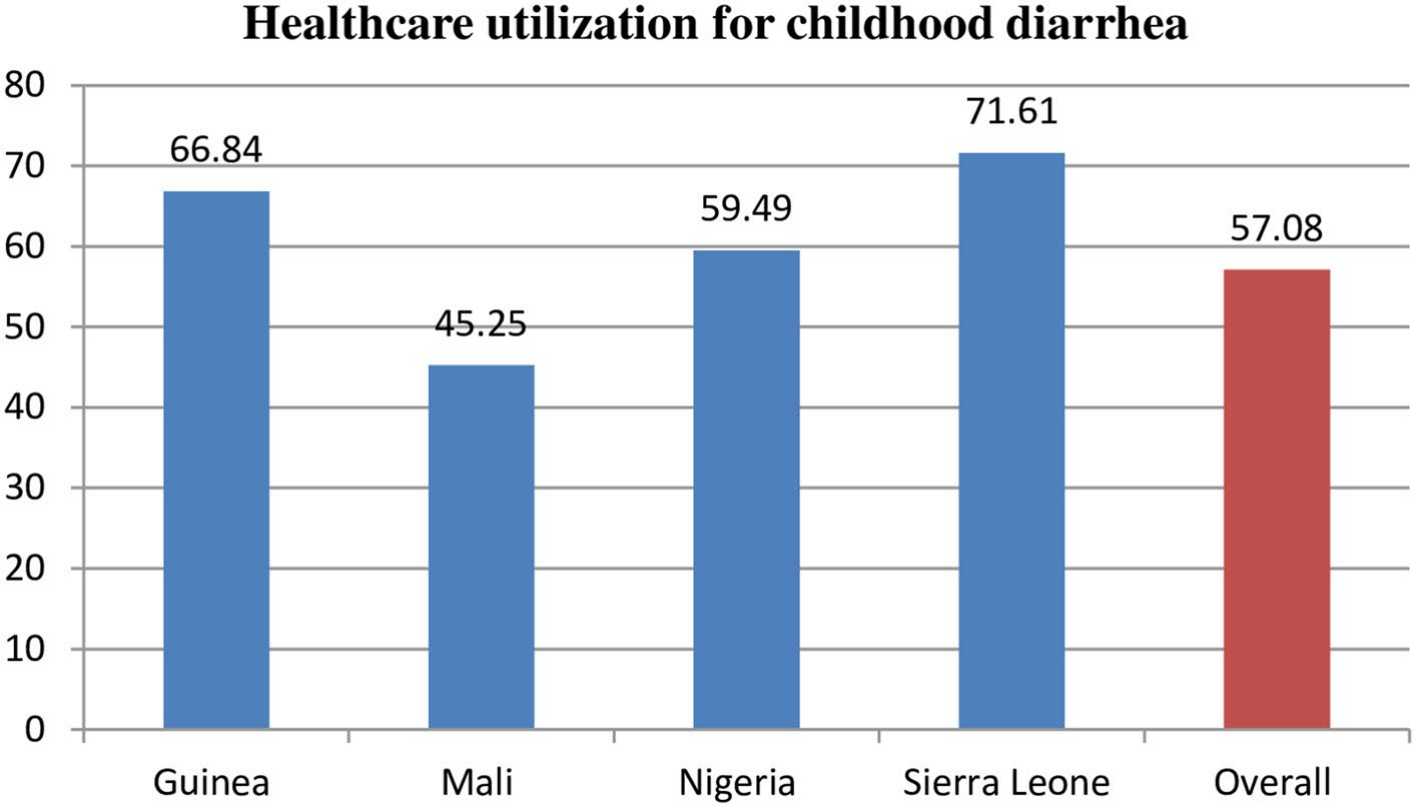
magnitude of healthcare utilization for childhood diarrhea in top high under-five mortality countries.

The overall utilization of healthcare for childhood diarrhea in countries with high under-five mortality was 58.40% (95% CI 57.26%, 59.53%). The highest magnitude of healthcare utilization was in Sierra Leone (71.61%) and the lowest magnitude of healthcare utilization for childhood diarrhea was in Mali (45.25%) (Fig. 2).

### Factors associated with healthcare utilization for childhood diarrhea in top high under-five mortality countries.

#### Fixed effects (measures of association) result

The third model was the complete model, which shows the association of individual and community-level factors of healthcare utilization for childhood diarrhea among mothers in top high under-five mortality countries. The husband’s educational status, household wealth index, media exposure, information about oral rehydration, place of delivery, number of living children, and country of residence were significant predictors of healthcare utilization for childhood diarrhea.

Accordingly, mothers who had husbands with primary education (AOR = 1.29; 95% CI 1.07, 1.56) and secondary or higher education (AOR = 1.28; 95% CI 1.09, 1.51) were 1.29 times and 1.28 times more likely to seek healthcare for childhood diarrhea than mothers with an uneducated husband, respectively. The likelihood of healthcare utilization for childhood diarrhea among mothers/caregivers from middle household wealth status increased by 21% (AOR = 1.21; 95% CI 1.03, 1.43) as compared to mothers/caregivers from poor household wealth status. Mothers/caregivers who had media exposure had 1.31 times (AOR = 1.31; 95% CI 1.15, 1.50) higher likelihood of seeking healthcare for childhood diarrhea as compared to their counterparts. Mothers/caregivers who had information about oral rehydration were twice (AOR = 2.09; 95% CI 1.77, 2.46) more likely to utilize healthcare for childhood diarrhea than their counterparts. Concerning place of delivery, mothers who delivered in a health facility were 1.26 times (AOR = 1.26; 95% CI 1.10, 1.44) more likely to utilize healthcare for childhood diarrhea than those who delivered at home. Additionally, mothers/caregivers who had 3 – 4 children and five or more children were 23% (AOR = 077; 95 CI% 0.62, 0.95) and 28% (AOR = 0.72; 95% CI 0.56, 0.94) less likely to utilize healthcare for childhood diarrhea as compared to mothers who had 1 – 2 children, respectively. Furthermore, the odds of utilizing healthcare for childhood diarrhea were 64% (AOR = 0.34; 95% CI 0.28, 0.42) and 34% (AOR = 0.66; 95% CI 0.54, 0.80) lower in Mali and Nigeria as compared to Guinea, respectively (Table 4).

**Table 4:** Multilevel analysis of factors associated with health care utilization for childhood diarrhea in top high under-five mortality countries (n = 7254).

Variables	Healthcare utilization		Null model	Model I AOR (95%CI)	Model II AOR (95%CI)	Model III AOR (95%CI)
	Utilized (n = 4236 ) (%)	Not utilized (n = 3018) (%)				
Sex of child						
Male	2,183 (58.59)	1,543 (41.41 )		1		1
Female	2053 (58.20)	1475 (41.80)		0.99 (0.89, 1.10)		0.99 (0.89, 1.10)
Age of the child						
Less than 12 months	940 (55.00)	770 (45.00)		1		1
≥ 12 months	3296 (59.45)	2248 (40.55)		1.28 (0.96, 1.31)		1.26 (0.93, 1.45)
Maternal age						
15–24 years	1305 (57.53)	964 (42.47)		1		1
25–34 years	2006 (59.48)	1367 (40.52)		1.06 (0.90, 1.25)		1.00 (0.85, 1.18)
35–49 years	925 (57.38)	687 (42.62)		0.99 (0.80, 1.23)		0.92 (0.72, 1.12)
Maternal educational status						
No education	2614 (56.69)	1997 (43.31)		1		1
Primary	648 (62.53)	388 (37.47)		1.01 (0.85, 1.20)		0.97 (0.84, 1.19)
Secondary & above	974 (60.64)	633 (39.36)		0.83 (0.69, 0.99)		0.83 (0.68, 1.00)
Husband’s educational status						
No education	2180 (54.49)	1821 (45.51)		1		1
Primary	475 (62.18)	289 (37.82)		1.31 (1.08, 1.57)		1.29 (1.07, 1.56)**
Secondary & above	1354 (63.77)	769 (36.23)		1.40 (1.20, 1.63)		1.28 (1.09, 1.52)**
Sex of household head						
Male	3769 (58.26)	2700 (41.74)		1		1
Female	467 (59.53)	318 (40.47)		1.06 (0.87, 1.30)		1.09 (0.89, 1.33)
Wealth index						
Poor	2124 (55.56)	1700 (44.44)		1		1
Middle	882 (62.02)	540 (37.98)		1.17 (1.00, 1.36)		1.21 (1.03, 1.43)*
Rich	1230 (61.25)	778 (38.75)		1.00 (0.84, 1.18)		1.11 (0.91, 1.36)
Media exposure						
Exposed	2775 (60.47)	1814 (39.53)		1.16 (1.03, 1.32)		1.32 (1.16, 1.51)***
Non-exposed	1461 (54.83)	1204 (45.17)		1		1
Information about oral rehydration						
Yes	3825 (61.45)	2400 (38.55)		2.33 (1.98, 2.74)		2.08 (1.77, 2.46)***
No	411 (39.95)	618 (60.05)		1		1
Covered by health insurance						
Yes	122 (69.92)	53 (30.08)		1.28 (0.87, 1.87)		1.42 (0.96, 2.08)
No	4114 (58.12)	2965 (41.88)		1		1
Wanted last-child						
Wanted	4135 (58.51)	2932 (41.49)		1.21 (0.85, 1.70)		1.26 (0.89, 1.80)
Unwanted	101 (54.20)	86 (45.80)		1		1
Place delivery						
At home	2190 (55.47)	1758 (44.53)		1		1
Health facility	2047 (61.90)	1260 (38.10)		1.22 (1.07, 1.38)		1.26 (1.10, 1.44)***
Birth order						
First	766 (57.76)	561 (42.24)		1		1
Second	734 (57.03)	553 (42.97)		0.99 (0.81, 1.19)		1.02 (0.84, 1.23)
Third	655 (60.33)	430 (39.67)		1.31 (0.92, 1.70)		1.34 (1.04, 1.75)
Fourth or above	2081 (58.55)	1474 (41.45)		1.41 (0.86, 1.88)		1.48 (1.14, 2.03)
Number of living children						
1_2 children	1581 (58.82)	1107 (41.18)		1		1
3_4 children	1383 (58.89)	966 (41.11)		0.75 (0.61, 0.93)		0.77 (0.62, 0.95)*
5 + children	1272 (57.38)	945 (42.62)		0.66 (0.52, 0.86)		0.72 (0.56, 0.94)*
Place of residence						
Urban	1209 (60.91)	776 (39.09)			1	1
Rural	3027 (57.46)	2242 (42.54)			1.05 (0.90, 1.23)	1.26 (0.99, 1.52)
Distance to the health facility						
Not big problem	2747 (59.60)	1862 (40.40)			1.15 (1.02, 1.29)	1.05 (0.92, 1.19)
Big problem	1489 (56.31)	1156 (43.69)			1	1
Community level education						
High	2158 (60.61)	1402 (39.39)			1.04 (0.87, 1.24)	1.02 (0.85, 1.24)
Low	2078 (56.27)	1616 (43.73)			1	1
Community level poverty						
High	2056 (56.53)	1581 (43.47)		1.13 (0.95, 1.34)	0.92 (0.76, 1.10)	1.00 (0.82, 1.23)
Low	2180 (60.28)	1437 (39.72)			1	1
Community-level media exposure						
High	2258 (59.14)	1561 (40.86)			1.13 (0.95, 1.34)	0.99 (0.83, 1.18)
Low	1978 (57.58)	1457 (42.42)			1	1
Country of residence						
Guinea	697 (66.84)	346 (33.16)			1	1
Mali	738 (45.25)	893 (54.75)			0.35 (0.29, 0.43)	0.34 (0.28, 0.42)***
Nigeria	2350 (59.49)	1600 (40.51)			0.63 (0.53, 0.75)	0.66 (0.54, 0.80)***
Sierra Leone	451 (71.61)	179 (28.39)			1.23 (0.96, 1.58)	1.10 (0.84, 1.44)
Measures of variations						
Variance			0.61 (0.49, 0.77)	0.59 (0.46, 0.75)	0.56 (0.45, 0.72)	0.54 (0.43, 0.71)
ICC (%)			15.75%	15.23%	14.72%	14.31%
MOR			2.02	1.98	1.93	1.90
PCV			Ref	4.9%	8.2%	11.5%
Model fitness						
Deviance (-2logliklihood)			9546.54	8874.34	9370.58	8731.68

The null model contains no explanatory variables; Model I includes individual-level factors only; Model II includes community-level variables only; Model III includes both individual and community-level factors, AOR: Adjusted odds ratio, CI: Confidence internal, ICC: Intra-class correlation coefficient, MOR: Median odds ratio, PCV: Proportional change in variance.

*: *p*-value less 0.05; **: *p*-value less than 0.01; ***: *p*-value less than 0.001.

### Random effect (measures of variation) result

The final model (model III) was the best-fitted model since it had the lowest deviance. The ICC in the null model was 15.75%, which revealed that about 15.75% of the total variability of healthcare utilization for childhood diarrhea was due to cluster differences. Moreover, the MOR was 2.02 in the null model, and this indicated that there was a variation between clusters. A mother/caregiver in the cluster with a high likelihood of utilizing healthcare for childhood diarrhea had twice higher odds of being utilizing healthcare compared with a mother/caregiver in a cluster with a low likelihood of healthcare utilization for childhood diarrhea during random selection of mothers/caregivers in two different clusters. The full model explained 11.5% of the variability in seeking healthcare for childhood diarrhea, and deviance was used for model fitness (Table 4).

## Discussion

This study aimed to determine the magnitude and identify the determinant factors of healthcare utilization for childhood diarrhea in top high under-five mortality countries. The result of this study showed that the magnitude of utilization of healthcare for childhood diarrhea in countries with high under-five mortality was 58.40%. Regarding the detrminants of healthcare utilization for childhood diarrhea; the husband's educational status, household wealth index, media exposure, information about oral rehydration, place of delivery, number of living children, and country of residence were identified as predictors of healthcare utilization for childhood diarrhea in high under five mortality countries.

The overall magnitude of healthcare utilization for childhood diarrhea in the top high under-five mortality countries was 58.40% (95% CI 57.26%, 59.53%), which is consistent with studies conducted in Ethiopia^17,26,32^. However, the finding of this study is higher than a study conducted in Ethiopia^6,15,23,33^, and Nigeria^14^. The possible explanation for the difference might be the study sample size, this study has a larger sample size than the previous studies conducted in the single study setting. In this regard, the larger sample size might lead to a high magnitude of healthcare utilization for childhood diarrhea in this study. Additonally, it is higher than a study done in SSA^16^, which might be due to the survey year differnces in which as the survey year increases the awareness to use modern healthcare for illness may inceases.

The current study finding showed a lower magnitude of healthcare utilization for childhood diarrhea than study reports in Ethiopia^9,12^ and Indonesia^28^. The discrepancy might be explained by the definition of the outcome variable, where the previous studies assessed healthcare utilization for common childhood illnesses, whereas this study focused only on childhood diarrhea.

This study revealed that mothers/caregivers who had primary or above-educated husbands were more likely to utilize healthcare for childhood diarrhea than mothers with uneducated husbands. This finding is consistent with studies done in Ethiopia^19,23,33^. The possible justification might be that education can be assumed to be related to an increased awareness of symptoms, illnesses, and the availability of services. Moreover, educational level is a major factor in higher employment opportunities, which may in turn increase healthcare utilization by enhancing the ability to cope with the various costs involved^23^. The finding indicates the government should strengthen education programs to improve healthcare utilization.

The odd of healthcare utilization for childhood diarrhea was high among mothers/caregivers of middle household wealth status as compared to poor household wealth status. Other studies in Ethiopia^12,18,19,23,24^, Bangladesh^21,22^, SSA^16^, Zimbabwe^34^ and Gambia^25^ also supported our findings. The possible reason might be that children might not get the required medical attention due to the mother’s inability to pay for health services from poor household wealth status. Media exposure was also found to be a positive predictor of healthcare utilization for childhood diarrhea, which is supported by studies done in Ethiopia^18^, SSA^16^, and Bangladesh^22^. This could be explained by media can be useful for the dissemination of health information and healthcare, which could enhance people's understanding, attitudes, and behaviors about the utilization of health services.

Oral rehydration information is also another determinant of healthcare utilization for childhood diarrhea. Mothers/caregivers who had information about oral rehydration had a higher likelihood of healthcare utilization for childhood diarrhea as compared to their counterparts. This study finding is consistent with studies done in Ethiopia^15,18^ and Gambia^25^. The possible explanation for this might be those mothers aware of the oral rehydration may go to the health facilities immediately to seek care for their children, as they may have a better understanding of oral rehydration treatment. This implies that awareness creation about oral rehydration through media and other strategies will improve healthcare utilization for childhood diarrhea.

Mothers/caregivers who gave their last birth in a health facility were more likely to utilize healthcare for childhood diarrhea than those who gave birth at home. This study finding is comparable with studies conducted in Nigeria^14^ and Ethiopia^6^. The possible explanation for this result might be that facility birth enable mothers to be aware of the advantages of seeking healthcare at the time of a child's illness. This finding implies the government needs to develop strategies to enhance health facility delivery.

Additionally, this study also found that the number of living children was negatively associated with maternal healthcare utilization for childhood diarrhea. This could be explained by mothers’ high workload due to large family size, could bring about giving less attention to the sick child. This finding contradicts the finding from Burundi^27^ which reported that children of mothers who had three and four children were more likely to get healthcare for childhood illnesses compared to those whose mothers had one child. The reason for the variation might be due to the definition of the outcome variable, where the previous study dealt with common childhood illnesses while our study was specifically focused on childhood diarrhea. Furthermore, the odds of utilizing healthcare for childhood diarrhea were lower in Mali and Nigeria as compared to Guinea. The possible reason might be due to the countries' differences in terms of their health systems, policies, government structures, and health institutions.

## Strengths and limitations of the study

The secondary data used for the analysis was extracted from a nationally representative survey collected by employing a two-stage stratified sampling technique. The analysis with multilevel models using confidence intervals helps to determine the cluster variation in the hierarchical data of DHS. However, the cross-sectional nature of the survey limits to establish a cause-and-effect relationship. Additionally, since the study used secondary data variables related to perceived severity of illness, ORS knowledge and distance to health facilities couldn’t be addressed. Measuring mothers’ perceptions of childhood illness may not always be true.

## Conclusion

More than four in ten children didn’t receive health care for childhood diarrhea in the top under-five mortality countries. Thus, to increase healthcare utilization for childhood diarrhea, health managers and policymakers should develop strategies to improve the household wealth status of those with poor household wealth indexes. The decision-makers and the government of these countries should increase access to education and work on mass media to sensitize women about oral rehydration and facility birth.

## Data Availability

Data used in our study are publicly available upon request from the DHS program website. (https://dhsprogram.com/).

## Abbreviations

AOR: Adjusted odds ratio
CI: Confidence interval
DHS: Demographic and health survey
ICC: Intra-class correlation coefficient
IQR: Interquartile range
LR: Likelihood ratio
MOR: Median odds ratio
PCV: Proportional change in variance
SDG: Sustainable development goal
SSA: Sub-Saharan Africa
